# The Genome Sequences of *Cellulomonas fimi* and “*Cellvibrio gilvus”* Reveal the Cellulolytic Strategies of Two Facultative Anaerobes, Transfer of “*Cellvibrio gilvus*” to the Genus *Cellulomonas*, and Proposal of *Cellulomonas gilvus* sp. nov

**DOI:** 10.1371/journal.pone.0053954

**Published:** 2013-01-14

**Authors:** Melissa R. Christopherson, Garret Suen, Shanti Bramhacharya, Kelsea A. Jewell, Frank O. Aylward, David Mead, Phillip J. Brumm

**Affiliations:** 1 Department of Bacteriology, University of Wisconsin-Madison, Madison, Wisconsin, United States of America; 2 Department of Energy, Great Lakes Bioenergy Research Center, University of Wisconsin-Madison, Madison, Wisconsin, United States of America; 3 Lucigen, Middleton, Wisconsin, United States of America; 4 C5-6 Technologies, Middleton, Wisconsin, United States of America; University of Florida, United States of America

## Abstract

Actinobacteria in the genus *Cellulomonas* are the only known and reported cellulolytic facultative anaerobes. To better understand the cellulolytic strategy employed by these bacteria, we sequenced the genome of the *Cellulomonas fimi* ATCC 484^T^. For comparative purposes, we also sequenced the genome of the aerobic cellulolytic “*Cellvibrio gilvus”* ATCC 13127^T^. An initial analysis of these genomes using phylogenetic and whole-genome comparison revealed that “*Cellvibrio gilvus”* belongs to the genus *Cellulomonas*. We thus propose to assign “*Cellvibrio gilvus”* to the genus *Cellulomonas*. A comparative genomics analysis between these two *Cellulomonas* genome sequences and the recently completed genome for *Cellulomonas flavigena* ATCC 482^T^ showed that these cellulomonads do not encode cellulosomes but appear to degrade cellulose by secreting multi-domain glycoside hydrolases. Despite the minimal number of carbohydrate-active enzymes encoded by these genomes, as compared to other known cellulolytic organisms, these bacteria were found to be proficient at degrading and utilizing a diverse set of carbohydrates, including crystalline cellulose. Moreover, they also encode for proteins required for the fermentation of hexose and xylose sugars into products such as ethanol. Finally, we found relatively few significant differences between the predicted carbohydrate-active enzymes encoded by these *Cellulomonas* genomes, in contrast to previous studies reporting differences in physiological approaches for carbohydrate degradation. Our sequencing and analysis of these genomes sheds light onto the mechanism through which these facultative anaerobes degrade cellulose, suggesting that the sequenced cellulomonads use secreted, multidomain enzymes to degrade cellulose in a way that is distinct from known anaerobic cellulolytic strategies.

## Introduction

The expanding development of biofuels has renewed interest in cellulose-degrading microorganisms. Cellulose is an attractive source for biofuel production for many reasons. As a component of plant cell walls, cellulose is the most abundant terrestrial source of carbon. Despite the huge biological presence of cellulose, relatively few organisms are capable of cellulose degradation, and those that have been described as cellulolytic primarily include bacteria and fungi; although cellulases have been isolated from Archaea [1] as well as higher Eukaryotes [2]. As a result, our current knowledge of the mechanisms involved in the degradation of cellulose is derived mostly from a handful of cellulolytic microorganisms. Characterization of additional microorganisms that degrade cellulose may reveal novel cellulolytic mechanisms or cellulases that could enhance industrial strategies for the conversion of cellulose into commercially relevant products.

Cellulose degradation (recently reviewed in [3]) by microbes can be divided into two distinct strategies. These include the ‘secreted enzyme’ strategies, where cellulases are released into the extracellular environment away from the cell, and the ‘surface enzyme’ strategies, where an organism uses surface-associated cellulases to degrade fiber near the cell surface. The secreted enzyme’ approach appears to be employed by a few bacterial phyla (reviewed in [4]) and are typically associated with aerobic organisms. For example, this strategy is used by two closely-related cellulolytic Gammaproteobacteria, *Saccharophagus degradans* and *Cellvibrio japonicus* [5], as well as in numerous Actinobacteria [6]. Genome sequences for these bacteria have advanced our understanding of their cellulolytic mechanisms [7], showing that both organisms secrete their entire repertoire of polysaccharide-degrading enzymes. These enzymes contain multiple carbohydrate-binding-modules coupled to cellulase domains, thereby ensuring substrate-specificity [7].

In contrast, the ‘surface enzyme’ approach to cellulose degradation is primarily used by anaerobic bacteria. Anaerobic bacteria generally require close contact with the cellulose fiber and have cell-associated cellulase enzymes [4]. For instance, cellulosomes, which are characterized by multi-domain cellulase enzymes anchored in a cell-attached scaffold, have been identified in many *Clostridium* and *Ruminococcus* species [8]. Though the strict anaerobe *Fibrobacter succinogenes* does not use a canonical cellulosome, cell contact with the cellulose fiber is required for this organism to degrade cellulose and its many cellulases and hemicellulases are thought to act synergistically [9].

It is not known if the ‘secreted enzyme’ and the ‘surface enzyme’ approaches to cellulose degradation are mutually exclusive, or why these approaches were adopted by physiologically distinct groups of microorganisms [4]. However, members of the genus *Cellulomonas* provide an exception to these strategies because they, along with *Actinotalea fermentans* (formerly *Cellulomonas fermentans* [10]), are the only known facultative anaerobes reported to degrade cellulose under both conditions [4]. Importantly, many *Cellulomonas* strains, including *C. uda*, *C.* sp. CS-1 and *C. flavigena* are reported to use a mixture of cell-free and cell-associated cellulases [11], [12].

To gain insights into how different genera of aerobic and anaerobic bacteria degrade cellulose, we sequenced the genomes of “*Cellvibrio gilvus”* and *Cellulomonas fimi* and compared their metabolic and cellulolytic strategies. Upon examination of the “*Cellvibrio gilvus*” genome sequence, we found that many of its genomic attributes did not correspond to Gammaproteobacteria genomes, including that of *Cellvibrio japonicus* [7]. Here, we provide multiple lines of evidence for the transfer of “*Cellvibrio gilvus”* into the genus *Cellulomonas.* Since the name “*Cellvibrio gilvus*” was not validly published using proper taxonomic protocol, we propose *Cellulomonas gilvus* sp. nov. (type strain ATCC 13127^T^) here.

In addition to the proposal of *Cellulomonas gilvus* sp. nov. and the sequencing of *Cellulomonas fimi*, we performed a comparative analysis of these two *Cellulomonas* genome sequences with the recently reported genome sequence of *Cellulomonas flavigena* [13]. The reported ability of cellulomonads to degrade cellulose under both aerobic and anaeorobic conditions presents the hypothesis that these organisms utilize different strategies based on condition. We tested this hypothesis by analyzing these three cellulomonad genomes, but found that any differences in cellulose degradation were not reflected by their genomes. Specifically, we did not find homologs of the typical cellulosome components (scaffoldins, dockerins, or cohesins) within any of the sequenced cellulomonads, despite reports of cell-associated cellulase activity and the formation of cellulosome-like structures in *C. flavigena.* We found that the predicted percentage of secreted carbohydrate-active enzymes (CAZymes) was very similar between all three cellulomonads, although the number of predicted CAZymes was limited compared to other cellulase-secreting bacteria. Despite the limited number of CAZymes, we found that these cellulomonads were proficient at degrading and utilizing a diverse set of carbohydrates, including crystalline cellulose, *in vitro*. Our analysis reveals that the cellulolytic strategies predicted from their genome sequences do not match current models for cellulose degradation in these bacteria. Based on their genome sequences, we propose that these cellulomonads employ a ‘secreted enzyme’ approach to cellulose degradation under both aerobic and anaerobic condition but questions remain about the mechanisms employed during conditions where cell-associated cellulase activity has been reported for these organisms.

## Materials and Methods

### Growth Conditions, DNA Extraction, Genome Sequencing, and Finishing

The type strains for *Cellulomonas fimi* ATCC 484^ T^ and “*Cellvibrio gilvus*” ATCC 13127^T^ were obtained from the American Type Culture Collection. Cultures were grown in YTP-2 medium [14], which contains (per liter) 2.0 g yeast extract, 2.0 g tryptone, 2.0 g sodium pyruvate, 1.0 g KCl, 2.0 g KNO_3_, 2.0 g Na_2_HPO_4_^.^7H_2_O, 0.1 g MgSO_4_, 0.03 g CaCl_2_, and 2.0 ml clarified tomato juice. For preparation of genomic DNA, 1 L cultures were grown from a single colony in YTP-2 medium at 30°C with shaking at 200 rpm and collected by centrifugation. The cell concentrate was lysed using a combination of SDS and proteinase K, and genomic DNA was isolated using a standard phenol/chloroform extraction followed by alcohol precipitation.

The genomes were sequenced at the DOE Joint Genome Institute (JGI) using a combination of Illumina [15] and 454 technologies [16]. An Illumina GAii shotgun library with reads of 376 Mb, a 454 Titanium draft library with average read length of 450–465 bases, and a paired end 454 library with average insert size of 16 Kb were generated for this genome. General aspects of library construction and sequencing performed at the JGI can be found at http://www.jgi.doe.gov/. Illumina sequencing data was assembled with VELVET [17], and the consensus sequences were shredded into 1.5 kb overlapped fake reads and assembled together with the 454 data. Draft assemblies were based on 385.5 Mb 454 draft data, and 454 paired-end data. Newbler assembly parameters are -consed -a 50 -l 350 -g -m -ml 20.

For “*C. gilvus*”, the initial assembly contained 226 contigs in 13 scaffolds. The initial 454 assembly was converted into a phrap assembly by making fake reads from the consensus, collecting the read pairs in the 454 paired end library. The Phred/Phrap/Consed software package (http://www.phrap.com) was used for sequence assembly and quality assessment [18], [19], [20] in the following finishing process. After the shotgun stage, reads were assembled with parallel phrap (High Performance Software, LLC). Possible mis-assemblies were corrected with gapResolution (Cliff Han, unpublished), Dupfinisher [21], or by sequencing PCR fragments by subcloning or transposon bombing (Epicentre Biotechnologies, Madison, WI). Gaps between contigs were closed by editing in Consed, by PCR and by Bubble PCR primer walks. A total of 774 additional reactions and 1 shatter library were necessary to close gaps and to raise the quality of the finished sequence. The completed genome sequence of “*C. gilvus*” is 3,526,441 bases, with an error rate less than 1 in 100,000 bp. The genome sequence and its annotations can be obtained through GenBank under accession CP002665.1.

The *C. fimi* genome was assembled in the same manner as “*C. gilvus*”. Illumina sequencing (340 Mb) and 454 Titanium (20 kb paired-end) were generated. A draft assembly based on 285.8 MB 454 sequence data and all paired end sequence was generated (Newbler parameters consed -a 50 -1 350 -g -m -ml 20) containing 75 contigs in 11 scaffolds. The Illumina data was then incorporated and gaps between contigs were closed by PCR and by Bubble PCR primer walks. A total of 546 additional reactions and 6 shatter libraries were necessary to close gaps and to raise the quality of the finished sequence. The completed genome sequence of *C. fimi* is 4,266,344 with an error rate less than 1 in 10,000 bp. The genome sequence and its annotations can be obtained through GenBank under accession CP002666.1.

### Genome Annotation

The genome sequences of “*Cellvibrio gilvus”* and *Cellulomonas fimi* were annotated at Oak Ridge National Laboratory using a standard annotation pipeline. This includes the application of a number of annotation programs including open reading frame prediction using Prodigal [22]; automated protein function prediction using protein domains (Pfam) [23], Swiss-Prot [24], TIGRFAMs [25], KEGG [26], Interpro [27], and COG [28]; metabolic reconstruction analysis using PRIAM [29]; signal peptide prediction using SignalP [30]; tRNA prediction using tRNAscan-SE [31]; and rRNA prediction using RNAmmer [32]. These annotations can be publicly accessed at the Integrated Microbial Genomes (IMG) database (http://img.jgi.doe.gov/cgi-bin/w/main.cgi).

### Bacterial Growth Assays

*C. fimi* and “*C. gilvus*” were grown in YTP-2 medium [14] with pyruvate omitted (YTP-2X) at 30°C in 5 mL cultures using a 5% inoculum of an overnight culture. Cultures were supplemented with a variety of carbon sources, including: xyloglucan, glucomannan, galactomannan, lichenan, *beta*-glucan, rhamnogalacturonan, arabinogalactan, polygalacturonic acid, curdlan, galactan, and arabinan, which were obtained from Megazyme International (Wicklow, Ireland). Other carbon sources used include arabinoxylan, starch, glucose, cellulose, CMC, fructose, mannose, galactose, L-arabinose, D-arabinose, xylose, cellobiose, maltose, sucrose, mellibiose, and raffinose that were purchased from Sigma Aldrich (St. Louis, MO). All carbohydrates were evaluated at 2.0 g/L. Polysaccharides were autoclaved in the medium, while monosaccharides were added aseptically after autoclaving to prevent formation of Maillard reaction products. After 72 hours, the optical density (A_595_) and pH of all cultures was measured.

### Phylogenetic Analysis

Two sets of phylogenetic trees were constructed based on an alignment generated using MUSCLE [33]. Phylogenetic trees were based on either the DNA sequence encoding the 16S rRNA gene or a set of 32 single-copy highly-conserved housekeeping genes: *dnaA, dnaG, EF-TU, frr, gyrB, infC, nusA, pyrG, recA, rplA, rplB, rplC, rplD, rplE, rplF, rplK, rplM, rplN, rplP, rplS, rplT, rpmA, rpoB, rpsB, rpsC, rpsE, rpsI, rpsJ, rpsK, rpsM, rpsS, and smpB* from the genome sequences of “*Cellvibrio gilvus*” ATCC 13127^T^, *Cellvibrio japonicus* NCIMB 10462^T^, *Cellulomonas flavigena* DSM 20109^T^, *Cellulomonas fimi* ATCC 484^T^, *Buetenbergia cavernae* DSM 1233^T^, *Azotobacter vinelandii* BAA-1303^T^ *Microcccus luteus* NCTC 2665^T^, *Pseudomonas putida* KT2440, and *Bacillus subtilis subsp. subtilis str.* 168. Each sequence set was aligned using Muscle as implemented in Mega5 v5.1. The trees were constructed using Bayesian analysis as implemented in the program MrBayes (v3.1) [34] (ngen = 1,000,000, chain = 4), with the resulting tree visualized using FigTree (v1.3.1) (http://tree.bio.ed.ac.uk/software/figtree/.).

### Ortholog Analysis

Sequences from the predicted open reading frames from *Cellulomonas fimi*, *Cellulomonas flavigena*, and “*Cellvibrio gilvus*” were combined into one file. Protein pairs and their similarity scores were identified using the OrthoMCL Algorithm [35] in a series of steps outlined as described in the OrthoMCL software version 2.0 guide. The protein pairs were clustered using the Markov Cluster Algorithm [36]. From each cluster, we chose one representative sequence per organism, based on the sequence that produced the highest aggregate blast bit score when blasted against every other sequence. The bit scores were used to eliminate paralogs. Using these reduced clusters of representative sequences, we counted the number of sequences that were unique and those that were shared between the organisms. Unique proteins were then annotated using the Kyoto Encyclopedia of Genes and Genomes (KEGG) database [26].

## Results

### General Features of the Genomes

The two genomes, each composed of a single circular chromosome, differed greatly in size (Table 1). At 3.5 Mb, the genome of “*Cellvibrio gilvus*” was 0.74 Mb smaller than *Cellulomonas fimi* and encoded almost 600 fewer predicted open reading frames (ORFs). The GC content of the “*C. gilvus*” genome (73.8%) was more similar to *C. fimi* (74.7%) and *C. flavigena* (74.3%) than to *Cellvibrio japonicus* (52%). “*Cellvibrio gilvus*” had 45 tRNAs for each of the 20 amino acids, the same number as the other sequenced cellulomonads; this is in contrast to *Cellvibrio japonicus* which had 48 tRNAs. Furthermore, “*Cellvibrio gilvus*” and the sequenced cellulomonads had a smaller average coding sequence length, ranging from 1,008–1,017 bp with a maximum gene size of 6.2–7.6 Kb, compared to the *Cellvibrio japonicus* genome, which had an average gene size of 1,097 bp and a maximum gene length of 14 Kb with one notable exception of a predicted protein coding sequence of 38 Kb.

**Table 1:** Statistical features of the genome sequences of Cellvibrio japonicus, Cellvibrio gilvus, Cellulomonas fimi, and Cellulomonas flavigena.

Statistic	*Cellvibrio japonicus*	*Cellvibrio gilvus*	*Cellulomonas fimi*	*Cellulomonas flavigena*
Genome Size	4,576,573	3,526,441	4,266,344	4,123,179
G+C Content	52	73.8	74.7	74.3
Predicted Coding Sequences	3,750	3,164	3,762	3,678
% Genome Coding	89.9	91	89.6	89.9
Avg Coding Sequence Length	1,097	1,014	1,017	1,008
Largest gene size	38,229	7,143	6,210	7,755
Smallest gene size	90	96	90	96
tRNAs	48	45	45	45
rRNA operons	3	2	3	3
GenBank Accession	CP000934	CP002665	CP002666	CP001964
**Predicted Carbohydrate Active Enzymes**				
Glycoside Hydrolases	126	81	109	88
Cellulasesα	19	9	11	12
Xylanasesβ	8	7	7	19
Curdlanasesγ	23	15	28	19
Carbohydrate Esterases	19	11	10	14
Pectate Lyases	14	0	6	2
Carbohydrate Binding Modules	111	46	55	74
Glycosyl Transferases	45	33	47	50

α Sum of sequences from GH5, GH6, GH9, GH48, and GH94 families.

β Sum of sequences from GH10, GH11, and GH30 families.

γ Sum of sequences from GH16, GH55, GH64 and GT2 families.

We compared the genome of “*Cellvibrio gilvus*” with the genomes of *Cellulomonas fimi*, *Cellulomonas flavigena*, and *Cellvibrio japonicus* to identify conserved synteny. Synteny plots were generated using the SEED webserver (http://www.theseed.org/wiki/Home_of_the_SEED) [37]. We found macrosynteny between “*C. gilvus*” and the other sequenced *Cellulomonas* species but no synteny between “*C. gilvus*” and *Cellvibrio japonicus* (Figure S1), further supporting the transfer of “*Cellvibrio gilvus*” to the *Cellulomonas* group.

### Phylogenetic Analysis

**Figure 1:**
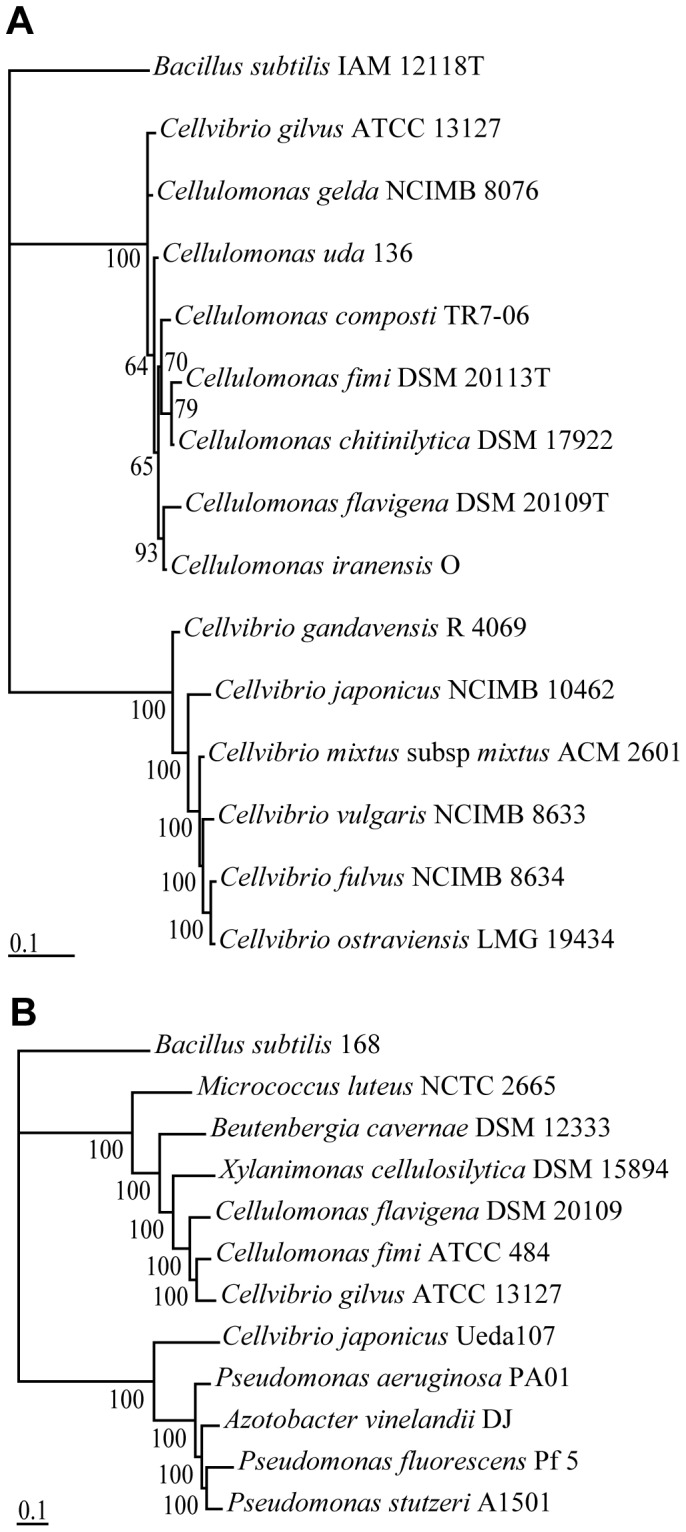
Phylogenetic placement of “*Cellvibrio gilvus*”. Rooted Bayesian trees based on 16S rRNA gene sequences (A) and 32 concatenated house-keeping protein sequences (B) showing the relationship between “*Cellvibrio gilvus”* and sequenced bacterial genomes in the phylum Actinobacteria and Gammaproteobacteria and species within the *Cellulomonas* and *Cellvibrio* genera. Bar, 0.1 substitutions per amino acid position.

To better resolve the phylogenetic placement of “*Cellvibrio gilvus*” we compared its genome to those of other members within the phyla Actinobacteria and Gammaproteobacteria. We first used the 16S rRNA gene from these genomes to construct a phylogenetic tree as shown in Figure 1A. Analysis of this tree shows that “*Cellvibrio gilvus*” grouped with species within the genus *Cellulomonas*, away from species of the genus *Cellvibrio*. To confirm this finding, we constructed a protein phylogenetic tree based on 32 single-copy highly-conserved housekeeping genes (Figure 1B). We found that “*Cellvibrio gilvus*” grouped most closely with *Cellulomonas fimi* and *Cellulomonas flavigena* within a cluster of sequenced organisms in the suborder Micrococcineae (phylum Actinobacteria). This cluster forms a phylogenetic line that is distinct from sequenced organisms such as *Cellvibrio japonicus* that are in the family Pseudomonadaceae (phylum Gammaproteobacteria) with 100% posterior probability.

### Gram Stain Analysis

The genus *Cellulomonas*, which belongs to the phylum Actinobacteria, are Gram-positive [38], [39]. To further confirm the identity of “*Cellvibrio gilvus*”, we scanned the “*Cellvibrio gilvus*” genome for genes involved in lipospolysaccharide biosynthesis and transport, a distinguishing feature between Gram-positive and Gram-negative organisms [40]. *Cellvibrio japonicus* served as a Gram-negative control, while *Cellulomonas flavigena and Cellulomonas fimi* served as Gram-positive controls. As expected, *Cellvibrio japonicus* contained a complete lipopolysaccharide biosynthetic pathway while *Cellulomonas flavigena, Cellulomonas fimi,* and “*Cellvibrio gilvus*” did not. In addition, the use of menaquinone or ubiquinone is also a distinguishing feature between Gram-negative and Gram-positive bacteria [41]. *Cellvibrio japonicus* was found to contain the complete pathway for synthesis of ubiquinone, as do most Gram-negative bacteria. In contrast, “*Cellvibrio gilvus*”, *Cellulomonas flavigena*, and *Cellulomonas fimi* encode for the menaquinone synthesis pathway instead of the ubiquinone synthetic pathway.

The original report describing “*Cellvibrio gilvus*” indicated that it is a Gram-negative organism [42]. However, it has been documented that the rate of Gram stain decolorization is very fast in species of the genus *Cellulomonas*, and thus can be misinterpreted as Gram-negative [39]. We performed a Gram stain on fresh cultures of “*Cellvibrio gilvus*” with a 20 second decolorization; *Escherichia coli* was used as a negative control and *Bacillus subtilis* as a positive control. Under these conditions, we found “*Cellvibrio gilvus*” to be Gram-positive (data not shown).

### Ortholog Analysis

**Figure 2:**
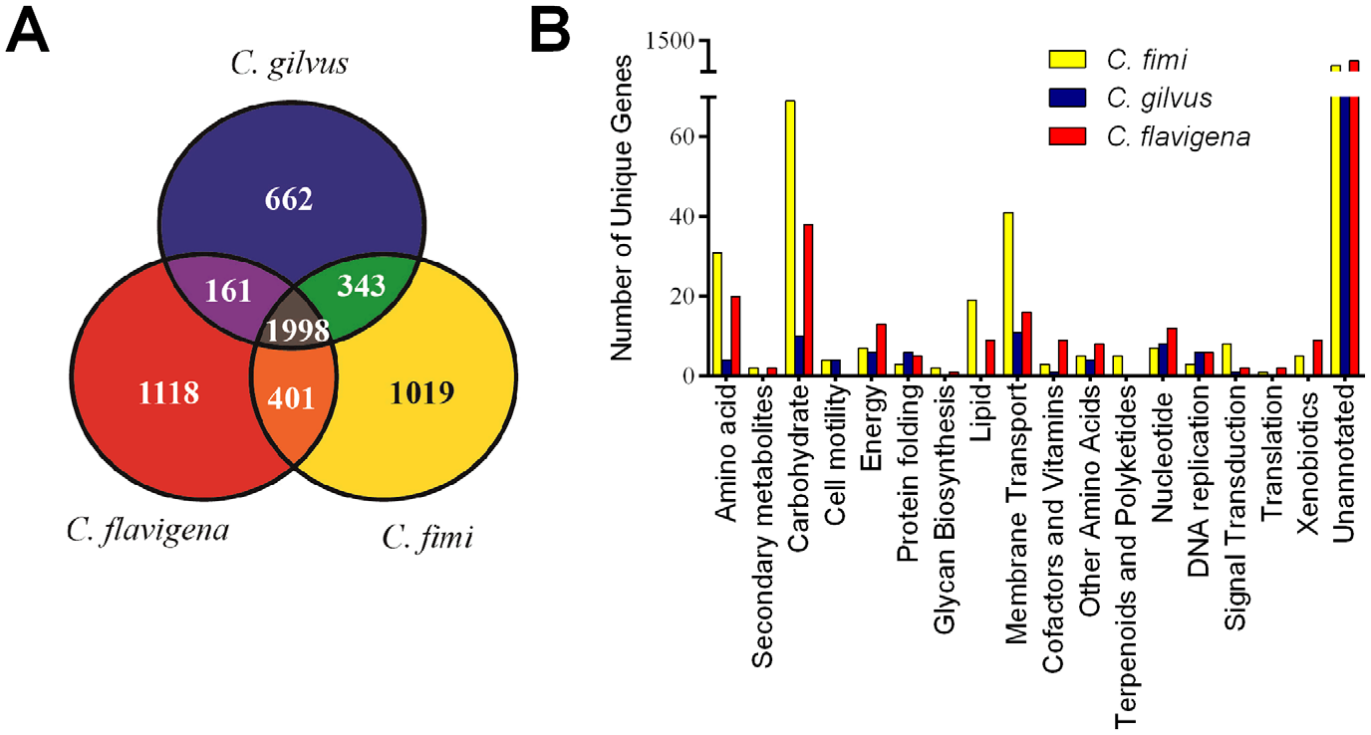
Ortholog analysis of the three *Cellulomonas* genomes conducted using OrthoMCL. The total numbers of shared proteins between the three genomes were tabulated and presented as a Venn diagram in (A). The unique proteins from each species were analyzed using the KEGG database (B).

Based on the analyses presented above, we propose that “*Cellvibrio gilvus*” be transferred to the *Cellulomonas* genus with the name *Cellulomonas gilvus* sp. nov. For the remainder of this study, we refer to this organism as *Cellulomonas gilvus*. To begin understanding the genomic similarities between the cellulomonads, we compared the genome of *C. gilvus* to those of *C. fimi* and *C. flavigena*. We first performed an OrthoMCL analysis to identify the set of orthologs shared between these bacteria (Figure 2A). We identified 1,998 orthologs shared between these cellulomonads and found that many of these encoded for housekeeping functions and central metabolism. Our analysis also identified coding sequence unique to each species, including 30% (1,118), 27% (1,019), and 21% (662) of the coding sequences within *C. flavigena*, *C. fimi*, and *C. gilvus*, respectively (Figure 2A). These numbers are likely an underestimation of the total unique proteins since paralogs are only counted once by OrthoMCL. *C. gilvus* had the fewest unique predicted proteins and shared twice as many predicted proteins with *C. fimi* than with *C. flavigena*. *C. fimi* had the most unique proteins that could be classified by annotation, many involved in carbohydrate metabolism and membrane transport, particularly propanoate metabolism, acetyl group transfer, and uronic acid interconversions (Figure 2B). Although *C. gilvus* has nearly 600 fewer predicted proteins than the other two sequenced cellulomonads, it lacked only 100 unique annotated functions (Table S1).

### Polysaccharide Metabolism

We characterized polysaccharide metabolism in *C. fimi* and *C. gilvus* by measuring growth and acid production from hemicelluloses. Both cultures produced acid (final culture pH <6.5) from glucomannan, galactomannan, arabinoxylan, starch, lichenan, *beta*-glucan, galactan, and glucose. In addition, *C. fimi* produced acid from arabinan while *C. gilvus* did not. Both cultures showed no acid production (final culture pH ≥8.0) but increased optical density (A_595_) when grown in medium supplemented with cellulose, xyloglucan, carboxymethyl cellulose (CMC), and rhamnogalacturonan. Acid was not produced (final culture pH ≥8.0) and measured optical density (A_595_) was not increased when the medium was supplemented with arabinogalactan, polygalacturonic acid, or curdlan.

### Physiological Comparison

To gain insight into the physiological properties of these three sequenced cellulomonads, we performed a physiological reconstruction analysis using the computer program PRIAM [29], which generates KEGG [26] maps. Despite the diverse phenotypes associated with members of the *Cellulomonas* genus, we found comparatively little variation between the genomes. Unique features of the morphology, carbohydrate utilization, and energy metabolism that influence cellulose degradation and utilization among the sequenced cellulomonads are highlighted in the following sections.

### Surface Structures

Surface structure and capsular polysaccharides have been implicated for their role in cellulose degradation in some *Cellulomonas* species. For instance, a “glycocalyx-like shell” was formed by *C. sp.* NRCC2406 when grown in the presence of cellulose; the “glycocalyx-like shell” was thought to contribute to fiber attachment and cellulose degradation [43]. Moreover, differences in surface structures such as flagella, cellulosome-like structures, and surface polysachcharides have been reported among *Cellulomonas* species.

We examined the genome sequences of our cellulomonads and found that many of these differences were not reflected in their genomes. For example, motility has been cited as an important factor in the cellulolytic strategy of some cellulomonads [44]. Despite one report of motility in *C. flavigena* ATCC 482^T^ (66), we found *C. flavigena* is the only sequenced cellulomonad of the three that lacked annotated flagellar genes. A KEGG analysis of the *C. fimi* and *C. gilvus* genomes predicts similar flagellar protein components, in agreement with their reported motility.

Curdlan (β-1-3-glucan) has also been proposed to play a role in fiber attachment for *C. flavigena* [44] and is produced abundantly by *C. flavigena* while *C. fimi* and other *Cellulomonas* species produce a small amount [45]. Curdlan, along with glycogen and trehalose have also been proposed as storage polysaccharides in *C. flavigena* [46]. All three organisms have 11 GH 13 family members that are intracellular and likely involved in glycogen synthesis, remodeling and degradation, or trehalose biosynthesis and degradation similar to that reported for *C. flavigena* [46]. The enzymes involved in curdlan synthesis are in the GT2 glycosyl transferase family but this family also functions in the synthesis of other oligosaccharides [47]. Each of the sequenced cellulomonads has numerous predicted GT2s: 19 in *C. flavigena*, 23 in *C. fimi*, and 15 in *C. gilvus* (Table S2). *Agrobacterium* and *Cellulomonas* are known to produce linear (1–3)-β-glucan and each sequenced cellulomonad has two GT2 enzymes with low sequence identity (35–39%) to *Agrobacterium* curdlan synthase (Cfla_3154 and _2615, Celf_3456 and _3585, Celgi_1506 and_0939). *C. fimi* was the only sequenced cellulomonad that contained predicted curdlanases GH16, GH55, and GH64 (Table 1), suggesting the ability to utilize curdlan. Based on the genome, it is unclear how *C. flavigena* reutilizes the curdlan it produces. In general, we found that all three cellulomonads contain similar numbers of curdlan synthesis genes.

Capsular polysaccharide production is linked to clusters of extracellular polysaccharide (EPS) genes, including transport, kinase and synthesis genes, in many organisms [48]. We identified a potential EPS gene cluster, containing UDP-N-acetylglucosamine, phosphoglycerate mutase, a regulator/membrane protein, histidine kinase, and a set of ATP-binding cassette (ABC) phosphor-transporters in the genomes of *C. fimi* and *C. gilvus* (Figure S2); however, a similar cluster was not found in *C. flavigena*. These findings may indicate differences in surface polysaccharides.

### Carbohydrate Utilization

To ascertain the carbohydrate degradation capacity of these cellulomonads, we performed a carbohydrate-active enzyme (CAZy) analysis. The sequenced cellulomonads appear to degrade cellulose and hemicelluloses using a limited number of CAZymes, roughly half of which are secreted. For those CAZymes that are secreted, Sec-dependent secretion is favored roughly 2∶1 over Twin-arginine-Translocase (TAT)-dependent secretion (Table S3), indicating that the majority of secreted CAZymes do not require intracellular folding or cofactors.

### Cellulose Utilization

Cellulomonads are known to degrade cellulose in both aerobic and anaerobic conditions. Since aerobes and anaerobes utilize different mechanisms for cellulose degradation, we looked for evidence of each strategy in the cellulomonas genomes. Anaerobic cellulose degradation is known to occur using the canonical cellulosome, cell-associated complexes of enzymes that facilitate cellulose degradation among fiber-attached cells. “Cellulosome-like” protuberances were reported to be formed on the surface of *C.* sp. ATCC 21399 in response to cellulose in the media [49]. Contact with the cellulose fiber was also found to be required for cellulose degradation in *C. gilvus* [50]. We found no evidence of traditional cellulosome components (*e.g*. dockerins, cohesins, or scaffoldins) in any of the *Cellulomonas* genomes. *C. gilvus* contains a single protein, Celgi_0311, which contains the LPXTG domain characteristic of cell-wall anchor proteins [51], [52]; no homologs to this protein were found in the other *Cellulomonas* genomes.

Analysis of predicted *endo*- and *exo*-cellulases encoded by the genomes of these cellulomonads revealed that degradation of cellulose is predicted to involve a maximum of 9 (*C. gilvus*), 11 (*C. fimi*) or 12 (*C. flavigena*) enzymes (Table S4); however, the actual numbers may be lower, since there is considerable CAZyme family overlap between cellulases, mannanases, curdlanases, and beta-glucanases. This is significantly less than the 19 and 31 potential cellulose-degrading enzymes predicted for the aerobic cellulose degrader *Cellvibrio japonicus* [7] and the prolific cellulose-degrading anaerobe *Fibrobacter succinogenes* S85 [9], respectively. The *Cellulomonas* species utilize a combination of GH5, GH6, GH9, and GH48 cellulases in addition to a single GH94 cellobiose phosphorylase. GH9 and GH48 cellulases have been shown to hydrolyze crystalline cellulose synergistically [53]. Most of the cellulases contain predicted signal peptides for secretion outside the cell. Carbohydrate binding module 2 (CBM2) domains, known to bind to crystalline cellulose, are found in all of the *Cellulomonas* cellulase families and are found in various combinations (Table S4). All three genomes also encode one or two GH9-CBM4-CBM4 combinations; CBM4 is known to bind to xylan, glucans, and amorphous cellulose, but not crystalline cellulose [54], suggesting a substrate other than crystalline cellulose for these enzymes.

We also identified a 4-gene operon with significant sequence similarity to the cellodextrin-utilization operon (*cld* operon) from *Bifidobacterium breve* [55] in each of the *Cellulomonas* genomes (Figure S3). We also identified loci in *C. gilvus* and *C. flavigen*a that had greater than 25% identity with the *Neurospora crassa* cellodextrin transporters NCU08114 and NCU00801. We did not find significant sequence similarity to the *cbp* cellodextrin transport/utilization operon [56] utilized by *Clostridium thermocellum*. *C. gilvus* has been demonstrated to transport cellodextrins as large as hexosaccharides and cleave them intracellularly [57]. Although cellobiose appears to be cleaved intracellularly by the cellobiose phosphorylase encoded by each of the cellulomonads, it is unclear what enzyme(s) could be involved in the intracellular cleavage of cellodextrins or similar polymers. There are several intracellular β-glucosidases in each of the sequenced genomes but none are known to hydrolyze cellodextrins. We found one intracellular cellulase (GH9) in *C. fimi* (Celf_1481) but could not identify any in *C. gilvus* or *C. flavigena*.

### Hemicellulose Utilization

In addition to cellulose utilization, cellulomonads are also known to actively degrade and metabolize hemicelluloses. We found that *C. fimi* and *C. gilvus* are more similar with respect to xylan degradation enzymes than *C. flavigena*. *C. fimi* and *C. gilvus* appear to use a combination of 7 extracellular and intracellular *endo*-xylanases while *C. flavigena* uses an unusual mixture of 19 exclusively extracellular *endo*-xylanases (Table S4). *C. gilvus* and *C. fimi* each encode a single intracellular GH10 with no signal peptide or CBM domain but also encode a number of multidomain secreted GH10s with different combinations of CBMs. In addition, *C. gilvus* and *C. fimi* also encode single secreted multi-functional GH11s and a single secreted GH30 xylanase. In contrast, *C. flavigena* encodes 12 extracellular multi-domain GH10s and 3 extracellular multi-domain GH11s in addition to several unique xylanases but no GH30 xylanases.

Further degradation of xylan to monosaccharides is accomplished using a combination of *beta*-xylosidases, *alpha*-arabinofuranosidases, and *alpha*-glucuronidases. All three organisms encode a combination of intracellular and extracellular GH43 *beta*-xylosidases that differ with respect to modularity; GH43 can occur as part of multidomain enzymes in *C. fimi* and *C. flavigena* but appears to occur singly in *C. gilvus*. *C. fimi* and *C. flavigena* also encode a single intracellular GH120 (predicted *beta*-xylosidase) and a single intracellular GH67 that *C. gilvus* lacks. All three cellulomonads encode an intracellular GH51 *alpha*-arabinofuranosidase and an extracellular GH62 *alpha*-arabinofuranosidase. However, only *C. fi*mi and *C. flavigena* encode a predicted intracellular *alpha*-glucuronidase.

### Other Carbohydrate Utilization and Interconversion

We also investigated these genome sequences for evidence of CAZymes involved in the degradation of other carbohydrates. We found that all three organisms possess a large number of GH13s (Table S2). Of these, only two in each organism appear to code for secreted *alpha*-amylases. *C. fimi* and *C. gilvus* each possess one xyloglucanase (GH74-CBM2) whereas *C. flavigena* has none. All three organisms also possess enzymes for the degradation of mannans (GH26 and GH113), *beta*-glucans (GH16 and GH81) and significant quantities of glycosyl transferases (GTs). *C. gilvus* and *C. flavigena* have similar numbers of GTs (47 and 48), while *C. fimi* has fewer (33). Many of these GT family members are involved in cell wall synthesis and the storage of polysaccharides and exopolysaccharides. In particular, GH39s and GH94s may be involved with glycosylation of secreted proteins.

We also identified differences in glucuronic acid interconversions between the *Cellulomonas* species. *C. fimi* and *C. flavigena* each contained multiple enzymes involved in uronic acid interconversions and pectate lyases that *C. gilvus* lacked (Table 1). *C. fimi* had six pectate lyases (PL) from the PL1, PL3, and PL11 families in addition to four gene products involved in uronic acid interconversions (Celf_3212, _3268, _3292, and _3346) while *C. flavigena* had a PL3 and a PL11 pectate lyase and six predicted uronic acid gene products (Cfla_0976, _2984, _3012, _0879, _9878, _3194). In contrast, *C. gilvus* had no predicted PL family members or gene products involved in uronic acid interconversions.

### Energy Metabolism and Fermentation

All three organisms can ferment hexoses and pentoses, therefore we examined the cellulomonad genomes for confirmation of the pathways of hexose and pentose fermentation. All three appear to possess complete Embden-Meyerhof pathways for the fermentation of hexose sugars as well as complete pentose phosphate pathways for the conversion of D-xylulose- and D-ribose-5-phosphate to pyruvate. However the genomes lack several isomerases that would allow broad pentose-sugar utilization, in agreement with our results and results published elsewhere. Specifically, each sequenced cellulomonad appears to encode enzymes for the fermentation of D-xylose but not ribitol, arabitol or arabinose, due to the lack of appropriate dehydrogenases and/or kinases. *C. flavigena* appears to be the only one of the three able to utilize ribose. Many cellulomonads are reported to show excellent growth under aerobic conditions and much reduced growth under anaerobic conditions, with all strains forming lactic and acetic acid from glucose [58], [59] while *C. uda* produced a mixture of formate, lactate, acetate, ethanol, and succinate from carbohydrates [60] when grown under anaerobic conditions.

The ability to re-utilize the products of fermentation differs among the cellulomonads. In contrast to the other two, *C. fimi* lacks an acetyl-CoA synthase gene explaining its inability to utilize acetate [39]. All three organisms encode at least one lactate dehydrogenase, as expected from reported lactate production by cellulomonads. The sequenced cellulomonads each encode a number of alcohol dehydrogenases (ADHs) indicating that they may be capable of ethanol production like *C. uda*. *C. fimi* encodes 15 ADHs, one which is iron-dependent ADH, and three which are zinc-dependent. *C. flavigena* encodes 10 ADHs including two that are iron-dependent. *C. gilvus* encodes six ADH, with one that is iron-dependent.

*C. gilvus* likely uses substrate level phosphorylation preferentially for aerobic respiration. *C. gilvus* lacks catalase and, in agreement with our growth analyses described above, has been reported to produce acid on many carbon sources, including cellobiose, glucose, sucrose and maltose. *C. gilvus* does not reduce nitrate [42], despite the presence of nitrate reductase genes, in contrast to *C. fimi*, *C. flavigena* and other cellulomonads which are known to reduce nitrate [39]. The inability to reduce nitrate may be due in part to insufficient uptake since these *Cellulomonas* species appeared to lack nitrate/nitrite transport system. Notably, *C. gilvus* encodes a predicted sulfate transport system while *C. fimi* and *C. flavigena* encodes an alkanesulfonate transport system. This could indicate a wider range of sulfur sources for *C. fimi* and *C. flavigena* when sulfate or cysteine are not available.

## Discussion

Here we present the complete genome sequences for *Cellulomonas fimi* and “*Cellvibrio gilvus*”. We provide multiple lines of evidence supporting the transfer of “*Cellvibrio gilvus*” to the genus *Cellulomonas,* including GC content (Table 1), phylogenetic analysis (Figure 1), synteny comparison (Figure S1), physiological characteristics, revisited Gram-stain, and analysis of LPS genes. Thus we propose *Cellulomonas gilvus* comb. nov. (type strain ATCC 13127^T^).

We compared the predicted proteome from all three sequenced *Cellulomonas* species, *C. fimi*, *C. flavigena*, and *C. gilvus* and found most functional enzyme classes were conserved across the three organisms despite the reduced genome size of *C. gilvus*. This broad, uniform reduction in genes from *C. gilvus* may indicate an efficient and streamlined organism (Figure 2). This reduction in the *C. gilvus* genome may suggest that this organism is in the process of reducing its genome, similar to obligate symbionts that require fewer genes to maintain a competitive existence. This is supported in part by the observation that *C. gilvus* has only ever been isolated from fresh ruminant feces [42], suggesting that it may be associated with the gastrointestinal tract of these animals. Moreover, *C. gilvus* is the only sequenced cellulomonad to lack catalase and, unusually, synthesizes CMP-N-acetylneuraminate based on the presence of N-acetylneuraminate synthase and citidylyltransferase (Celgi_1077 and _1078, respectively) (Table S1). CMP-N-acetylneuraminate is found on eukaryotic cell surfaces and is a component of the capsular polysaccharide of some bacterial pathogens [61]. This may indicate a host-associated niche or a unique exopolysaccharide composition for *C. gilvus*.

We found that the main genomic differences between the sequenced *Cellulomonas* species were related to surface structures and extracellular polysaccharides, including differences in motility and glucuronic acid interconversions. These predicted differences in surface structure may influence cellulolytic strategy. One proposed model suggested that secreted cellulases are sequestered near the cell surface by capsular polysaccharide [44]. This is supported by studies on *Cellulomonas flavigena* where >95% of CMC-dependent cellulase activity was found near the cell surface [44]. Surface structures have been correlated with cellulose metabolism in some *Cellulomonas* species [43], [49] and the importance of cell contact with the cellulose fiber has been debated [43], [50]. However, we found little evidence for cell-associated cellulases in the *Cellulomonas* genomes. Our analysis of the CAZymes suggests that each of the sequenced cellulomonads degrade cellulose and hemicelluloses using a limited number of multi-domain glycoside hydrolases, roughly half of which are predicted to be secreted (Table S3).

We found that all GH5, GH6, GH9, and GH48 cellulases are predicted to be secreted with the exception of one GH9 cellulase in *Cellulomonas fimi*. Furthermore, soluble cellulases and hemicellulases have been isolated and characterized from *C. fimi* [62], [63], [64], [65], [66], [67], [68], *C. flavigena* [59], [69], [70], [71] and *C. gilvus* [72], [73]. A reconstituted set of *C. fimi* cellulases were shown to effectively degrade cellulose [74], indicating cellular contact with substrate is not required for effective cellulose degradation by *C. fimi*. *C. gilvus* and *C. flavigena* possess homologues to these cellulases, and it is likely that these homologs confer similar properties. As a result, reports correlating cellulolytic strategy with differences in surface polysaccharide or surface structures are not substantiated by our genomic analysis; however it is possible that transcriptional differences account for variability seen in reported phenotypes or that hypothetical proteins or other unidentified components facilitate surface-associated cellulases in the cases where thos phenotypes have been observed.

Many of the *Cellulomonas* cellulases are part of multi-domain proteins containing carbohydrate binding moities. For example, two such multi-domain proteins identified in *C. flavigena*, CBP105 (Cfla_0016) and Cfla_0139 [6], [75] have structural characteristics similar to the *Thermomonospora fusca* processive endoglucanase Cel9A [76]. A processive endo-glucanase was also identified in *C. fimi*, CenC (celf_0019) [68]. We identified an enzyme with similar modules in *C. gilvus* (celgi_0019). Previous reports show that GH9 can act as both an endo- and an exo-cellulase and releases cellotetraose products in *T. fusca* [76]. The *Cellulomonas* species are predicted to transport cellobiose and hydrolyze it to glucose intracellularly. We identified a putative cellobiose transport operon in each of the sequenced cellulomonads (Figure S3B) that was recently shown to be common among Actinobacteria [6]. The possibility that this transporter could also transport cellodextrins, such as those released by the processive endo-cellulases, remains an interesting question.

The evidence presented by our genomic analysis does not support the hypothesis that cellulomonads use different strategies to degrade cellulose aerobically vs. anaerobically. We found no evidence for the typical ‘surface enzymes’ utilized by anaerobic cellulose degraders like cellulosomes. Our CAZy analysis also revealed that the cellulases encoded by these cellulomonads are strikingly similar to the ‘secreted enzyme’ approach employed by aerobic cellulose degraders. Taken together, we propose that these cellulomonads utilize the same approach to degrade cellulose in either aerobic or anaerobic conditions: the secretion of a specific set of cellulases into the extracellular media, though it is possible that unidentified components could facilitate a surface-enzyme strategy for cellulose decomposition under some conditions.

The process by which the cellulomonads degrade cellulose could inform industrial strategies for the conversion of cellulosic biomass to fuel. Several aspects of the *Cellulomonas* genomes indicate characteristics that make them attractive as a potential platform for biofuel production. They all appear to secrete relatively small number of enzymes capable of degrading cellulose and a range of hemicellulosic substrates. They also appear to be able to naturally ferment xylose and glucose sugars. The ability to ferment arabinose, arabitol, or ribose could potentially be conferred by the introduction of single genes encoding ribulose kinase, arabitol dehydrogenase, or ribokinase, respectively, *in trans*.

The number of ADH genes in *C. fimi* alone outnumbers the ADHs of the ethanologenic *Zymomonas mobilis* and *C. thermocellum* combined. Moreover, ethanol has been identified as a major fermentation product for some *Cellulomonas* strains [58], [60]. The *Cellulomonas* species do not encode pyruvate decarboxylase (PDC), an enzyme essential for homoethanol production in prominent ethanologenic organisms like *Zymomonas mobilis* and *Saccharomyces cereviciae*, however they do possess a pathway for ethanol production that is typical for bacterial species. This pathway utilizes pyruvate formate lyase to make acetyl-CoA and formate from pyruvate. Acetyl-CoA is converted to acetaldehyde by an Fe-dependent alcohol dehydrogenase and to ethanol by alcohol dehydrogenase. However, ethanol production by *Cellulomonas* species is likely not efficient since this pathway also results in the production of acetate and other fermentation products. This raises the interesting question about the potential use of a *Cellulomonas* species as a model for consolidated bioprocessing.

### Description of *Cellulomonas gilvus* sp. nov

***Cellulomonas gilvus*** (gil’vus. L. masc. adj. *gilvus* pale yellow-coloured).

Isolated exclusively from fresh bovine feces, cells with a size range of 0.75 to 1.5 by 1.5 to 3.75 μ are “straight to slightly curved rods with rounded ends, occuring singly, in pairs, and occasionally in short chains” when cultured on cellulose agar at 30°C while cell morphology on cellobiose media is variable [42]. Colonies on cellulose-yeast extract agar are slightly raised, convex to flat, opaque and white to pale yellow [42]. Gram-positive, motile, mesophilic, facultative anaerobe with G+C DNA content of 74.5% and optimal growth at neutral pH. Catalase-negative, indole, H_2_S, and acetylmethylcarbionol are not produced, nitrite is not released from nitrate, and ammonium sulfate does not serve as sole N source [42]. Acid is produced from glucomannan, galactomannan, arabinoxylan, starch, lichenan, *beta*-glucan, galactan, sucrose, maltose, cellobiose, and glucose, though cells contain no phosphoglucomutase. Growth, but no acid, is produced from mannose, galactose, starch, fructose, lactose, arabinan, xyloglucan, carboxymethyl-cellulose, rhamnogalacturonan, and cellulose, though genome does not encode cannonical cellulosome components. No growth is observed on arabinogalactan, polygalacturonic acid, ribose, glucuronic acid, methylglucose, or curdlan.

The type strain is ATCC 13127^T^.

## Supporting Information

Figure S1 **“** ***Cellvibrio gilvus*** **” shares macrosynteny with** ***Cellulomonas*** **species.** Synteny plot of “*Cellvibrio gilvus*” compared with other *Cellulomonas* species and *Cellvibrio japonicus* were generated using the SEED webserver (http://www.theseed.org/wiki/Home_of_the_SEED) [37]. Genes found in the two compared organisms are represented as dots on a graph where each axis indicates the gene position on the respective chromosome with the origin-of-replication at the x-y intercept.
(DOC)

Figure S2 **Potential EPS gene cluster in** ***C. gilvus*** **(Celgi_) and** ***C. fimi*** **(Celf_).** Genes are identified by respective gene number and color-coded according to predicted function: Green =  UDP-N-acetylglucosamine, Red = phosphoglycerate mutase, Blue = regulator/membrane protein,Yellow = Histidine kinase, Purple = ABC phospho- transporters. No significant match to this cluster was found in the *C. flavigena* genome.
(DOC)

Figure S3 **Predicted polysaccharide transport genes in the sequenced cellulomonads.** A) Homologs to proteins involved in polysaccharide transport identified in *Cellulomonas* species. B) Cellodextrin transport/utilization operon from *Bifidobacterium breve* (Bbr_) shares homology with operons identified in *Cellulomonas flavigena* (Cfla_), *Cellulomonas gilvus* (Celgi_), and *Cellulomonas fimi* (Celf_). Blocks indicate open reading frames that are labeled according to loci number or by gene name, where given. Operons are not drawn to scale. Hash marks indicate a distant position on the chromosome. Percent identity of each loci to the *B. breve* homolog is indicated.
(DOC)

Table S1 **Unique genes and their annotations from each sequenced cellulomonad.**
(DOC)

Table S2 **Glycoside Hydrolase and Glycosyl Transferase families of the sequenced cellulomonads.**
(DOC)

Table S3 **Secreted CAZymes in the sequenced cellulomonads.**
(DOC)

Table S4 **Cellulases, Xylanases, and hemicellulases of the sequenced cellulomonads.**
(DOC)
