# Supplementary material for: The Genome Sequences of Cellulomonas fimi and “Cellvibrio gilvus” Reveal the Cellulolytic Strategies of Two Facultative Anaerobes, Transfer of “Cellvibrio gilvus” to the Genus Cellulomonas, and Proposal of Cellulomonas gilvus sp. nov
Source: PLoS One. 2013 Jan 14;8(1):e53954. doi: 10.1371/journal.pone.0053954 (PMC3544764; doi:10.1371/journal.pone.0053954)
Supplement: Figure S1 — “ Cellvibrio gilvus ” shares macrosynteny with Cellulomonas species. Synteny plot of “Cellvibrio gilvus” compared with other Cellulomonas species and Cellvibrio japonicus were generated using the SEED webserver (http://www.theseed.org/wiki/Home_of_the_SEED) [37]. Genes found in the two compared organisms are represented as dots on a graph where each axis indicates the gene position on the respective chromosome with the origin-of-replication at the x-y intercept. (DOC) [file pone.0053954.s001.doc]

**Figure S1. Synteny between *Cellulomonas* and *Cellvibrio* species**.
