# Supplementary material for: The Genome Sequences of Cellulomonas fimi and “Cellvibrio gilvus” Reveal the Cellulolytic Strategies of Two Facultative Anaerobes, Transfer of “Cellvibrio gilvus” to the Genus Cellulomonas, and Proposal of Cellulomonas gilvus sp. nov
Source: PLoS One. 2013 Jan 14;8(1):e53954. doi: 10.1371/journal.pone.0053954 (PMC3544764; doi:10.1371/journal.pone.0053954)
Supplement: Figure S2 — Potential EPS gene cluster in C. gilvus (Celgi_) and C. fimi (Celf_). Genes are identified by respective gene number and color-coded according to predicted function: Green = UDP-N-acetylglucosamine, Red = phosphoglycerate mutase, Blue = regulator/membrane protein,Yellow = Histidine kinase, Purple = ABC phospho- transporters. No significant match to this cluster was found in the C. flavigena genome. (DOC) [file pone.0053954.s002.doc]

**Figure S2. Potential EPS gene cluster in *C. gilvus* and *C. fimi.***

Celgi_

|  | 0383 | 0384 | 0386 | 0387 | 0388 | 0389 | 0390 | 0391 |  |
| --- | --- | --- | --- | --- | --- | --- | --- | --- | --- |
|  |  |  |  |  |  |  |  |  |  |
| 0584 | 0585 | 0587 | 0588 | 0589 | 0590 | 0591 | 0592 | 0593 | 0594 |

Celf_
