# Supplementary material for: The Genome Sequences of Cellulomonas fimi and “Cellvibrio gilvus” Reveal the Cellulolytic Strategies of Two Facultative Anaerobes, Transfer of “Cellvibrio gilvus” to the Genus Cellulomonas, and Proposal of Cellulomonas gilvus sp. nov
Source: PLoS One. 2013 Jan 14;8(1):e53954. doi: 10.1371/journal.pone.0053954 (PMC3544764; doi:10.1371/journal.pone.0053954)
Supplement: Figure S3 — Predicted polysaccharide transport genes in the sequenced cellulomonads. A) Homologs to proteins involved in polysaccharide transport identified in Cellulomonas species. B) Cellodextrin transport/utilization operon from Bifidobacterium breve (Bbr_) shares homology with operons identified in Cellulomonas flavigena (Cfla_), Cellulomonas gilvus (Celgi_), and Cellulomonas fimi (Celf_). Blocks indicate open reading frames that are labeled according to loci number or by gene name, where given. Operons are not drawn to scale. Hash marks indicate a distant position on the chromosome. Percent identity of each loci to the B. breve homolog is indicated. (DOC) [file pone.0053954.s003.doc]

**Supplementary Figure 3**. Homologs of cellodextrin transporters in *Cellulomonas* species.

A

|  | *Cellulomonas gilvus* | | *Cellulomonas flavigena* | | *Cellulomonas fimi* | |
| --- | --- | --- | --- | --- | --- | --- |
| Query protein | Homolog | Score^*^ | Homolog | Score^*^ | Homolog | Score^*^ |
| CldR^1^ | Celgi_1052 | 196 | Cfla_2402 | 193 | Celf_1287 | 200 |
| CldG^1^ | Celgi_1053 | 257 | Cfla_2401 | 286 | Celf_1288 | 297 |
| CldF^1^ | Celgi_1054 | 340 | Cfla_2400 | 340 | Celf_1289 | 345 |
| CldE^1^ | Celgi_1055 | 300 | Cfla_2399 | 333 | Celf_1290 | 337 |
| CldC^1^ | Celgi_2447 | 498 | Cfla_1084 | 469 | Celf_2783 | 488 |
| CbpA^2^ | - | - | - | - | - | - |
| CbpB^2^ | Celgi_2245 | 88.6 | Cfla_0501 | 88.2 | Celf_1347 | 88.6 |
| CbpC^2^ | - | - | - | - | - | - |
| CbpD^2^ | - | - | - | - | - | - |
| lbp^2^ | Celgi_2865 | 128 | Cfla_1020 | 130 | Celf_2865 | 128 |
| NCU08114^3^ | Celgi_2645 | 105 | Cfla_2820 | 105 | - | - |
| NCU00801^3^ | Celgi_2645 | 117 | Cfla_2820 | 112 | - | - |

^*^ Score= the NCBI BLAST bit score indicates the strength of the alignment.

^1^ From *Bifidobacterium breve* UCC2003.

^2^ From *Clostridium thermocellum* ATCC27405.

^3^ From *Neurospora crassa* OR74A.

B
