# Supplementary material for: The Genome Sequences of Cellulomonas fimi and “Cellvibrio gilvus” Reveal the Cellulolytic Strategies of Two Facultative Anaerobes, Transfer of “Cellvibrio gilvus” to the Genus Cellulomonas, and Proposal of Cellulomonas gilvus sp. nov
Source: PLoS One. 2013 Jan 14;8(1):e53954. doi: 10.1371/journal.pone.0053954 (PMC3544764; doi:10.1371/journal.pone.0053954)
Supplement: Table S1 — Unique genes and their annotations from each sequenced cellulomonad. (DOC) [file pone.0053954.s004.doc]

**Table S1**. **Unique genes and their annotations from each sequenced cellulomonad.** Some annotations indicate similar functions but are derived from non-homologous proteins (*ie*: Celf_2562 and Cfla_3381).

| ***C. fimi*** | | ***C. flavigena*** | | ***C. gilvus*** | |
| --- | --- | --- | --- | --- | --- |
| gene | annotation | gene | annotation | gene | annotation |
| Celf_0682 | lactaldehyde reductase | Cfla_0081 | glucose-6-phosphate 1-dehydrogenase (EC:1.1.1.49) | Celgi_0155 | ribose-phosphate pyrophosphokinase (EC:2.7.6.1) |
| Celf_0730 | nucleotide sugar dehydrogenase (EC:1.1.1.22) | Cfla_0185 | ribose 5-phosphate isomerase | Celgi_0637 | beta-galactosidase (EC:3.2.1.21) |
| Celf_0885 | beta-galactosidase (EC:3.2.1.23) | Cfla_0318 | Phosphoglycerate mutase | Celgi_0766 | alpha amylase catalytic protein |
| Celf_0886 | beta-galactosidase (EC:3.2.1.23) | Cfla_0876 | carbohydrate kinase, thermoresistant glucokinase family (EC:2.7.1.12) | Celgi_1077 | N-acetylneuraminate synthase (EC:2.5.1.56) |
| Celf_1310 | acylphosphatase | Cfla_0879 | Glucuronate isomerase (EC:5.3.1.12) | Celgi_1078 | acylneuraminate cytidylyltransferase |
| Celf_1408 | phosphotransferase system EIIB/cysteine, phosphorylation protein | Cfla_1098 | alpha-glucan phosphorylase (EC:2.4.1.1) | Celgi_1856 | Lactate/malate dehydrogenase |
| Celf_1409 | phosphotransferase system EIIB/cysteine, phosphorylation protein | Cfla_1129 | glycoside hydrolase family 3 domain protein | Celgi_2131 | Haloacid dehalogenase domain-containing protein hydrolase |
| Celf_2053 | glycoside hydrolase family 3 domain-containing protein | Cfla_1381 | acetate/CoA ligase | Celgi_3088 | phosphoglycerate mutase |
| Celf_2283 | alpha amylase catalytic region | Cfla_1453 | Haloacid dehalogenase domain protein hydrolase |  |  |
| Celf_2550 | 2-methylcitrate dehydratase (EC:4.2.1.79) | Cfla_1916 | succinate dehydrogenase and fumarate reductase iron-sulfur protein |  |  |
| Celf_2551 | methylisocitrate lyase | Cfla_1917 | succinate dehydrogenase or fumarate reductase, flavoprotein subunit (EC:1.3.99.1) |  |  |
| Celf_2552 | 2-methylcitrate synthase/citrate synthase II | Cfla_1918 | succinate dehydrogenase (or fumarate reductase) cytochrome b subunit, b558 family |  |  |
| Celf_2555 | HpcH/HpaI aldolase | Cfla_2116 | oxidoreductase alpha (molybdopterin) subunit |  |  |
| Celf_2561 | pyruvate dehydrogenase E1 component subunit alpha (EC:1.2.4.1) | Cfla_2366 | NAD-dependent epimerase/dehydratase |  |  |
| Celf_2562 | transketolase central region | Cfla_2367 | GDP-mannose 4,6-dehydratase |  |  |
| Celf_2563 | hypothetical protein | Cfla_2836 | Alcohol dehydrogenase GroES domain protein |  |  |
| Celf_2564 | 3-oxoacid CoA-transferase subunit A (EC:2.8.3.5) | Cfla_2912 | 1,4-beta cellobiohydrolase |  |  |
| Celf_2565 | 3-oxoacid CoA-transferase subunit B (EC:2.8.3.5) | Cfla_3170 | Glycosyl hydrolase family 32 domain protein |  |  |
| Celf_2566 | acetyl-CoA acetyltransferase (EC:2.3.1.9) | Cfla_3198 | carbohydrate kinase, thermoresistant glucokinase family (EC:2.7.1.12) |  |  |
| Celf_2629 | acetyl-CoA acetyltransferase (EC:2.3.1.16) | Cfla_3381 | Transketolase central region |  |  |
| Celf_2718 | alpha-galactosidase (EC:3.2.1.22) | Cfla_3405 | Beta-fructofuranosidase (EC:3.2.1.26) |  |  |
| Celf_2983 | glycoside hydrolase family 3 domain-containing protein | Cfla_3595 | Nucleotidyl transferase |  |  |
| Celf_2991 | FMN-dependent alpha-hydroxy acid dehydrogenase | Cfla_3599 | NAD-dependent epimerase/dehydratase |  |  |
| Celf_3001 | acetyl-CoA acetyltransferase (EC:2.3.1.9) | Cfla_3603 | Transaldolase (EC:2.2.1.2) |  |  |
| Celf_3004 | 3-oxoacid CoA-transferase subunit B (EC:2.8.3.5) | Cfla_3643 | inositol monophosphatase |  |  |
| Celf_3005 | 3-oxoacid CoA-transferase subunit A (EC:2.8.3.5) |  |  |  |  |
| Celf_3013 | Rhamnulose-1-phosphate aldolase (EC:4.1.2.19) |  |  |  |  |
| Celf_3164 | PfkB domain-containing protein |  |  |  |  |
| Celf_3275 | glycoside hydrolase family 3 domain-containing protein |  |  |  |  |
| Celf_3287 | Altronate dehydratase (EC:4.2.1.7) |  |  |  |  |
| Celf_3306 | carbohydrate kinase |  |  |  |  |
| Celf_3313 | beta-galactosidase (EC:3.2.1.23) |  |  |  |  |
| Celf_3346 | 4-deoxy-L-threo-5-hexosulose-uronate ketol-isomerase (EC:5.3.1.17) |  |  |  |  |
| Celf_3472 | glycoside hydrolase family 3 domain-containing protein |  |  |  |  |
| Celf_3526 | glucose-6-phosphate dehydrogenase |  |  |  |  |
| Celf_3758 | class II aldolase/adducin family protein |  |  |  |  |
