# Supplementary material for: The Genome Sequences of Cellulomonas fimi and “Cellvibrio gilvus” Reveal the Cellulolytic Strategies of Two Facultative Anaerobes, Transfer of “Cellvibrio gilvus” to the Genus Cellulomonas, and Proposal of Cellulomonas gilvus sp. nov
Source: PLoS One. 2013 Jan 14;8(1):e53954. doi: 10.1371/journal.pone.0053954 (PMC3544764; doi:10.1371/journal.pone.0053954)
Supplement: Table S2 — Glycoside Hydrolase and Glycosyl Transferase families of the sequenced cellulomonads. (DOC) [file pone.0053954.s005.doc]

**Table S2. Glycoside Hydrolase and Glycosyl Transferase families of the sequenced cellulomonads.**

|  | ***C. gilvus*** | ***C. fimi*** | ***C. flavigena*** |
| --- | --- | --- | --- |
| **Glycoside Hydrolases** |  |  |  |
| GH13 | 17 | 18 | 14 |
| GH74 | 1 | 1 | 0 |
| GH16 | 0 | 3 | 0 |
| GH81 | 2 | 2 | 2 |
| GH26 | 1 | 3 | 3 |
| GH113 | 1 | 1 | 0 |
| **Glycosyl Transferases** |  |  |  |
| GT1 | 2 | 2 | 3 |
| GT2 | 23 | 15 | 19 |
| GT4 | 12 | 8 | 19 |
| GT9 | 2 | 0 | 0 |
| GT20 | 1 | 1 | 1 |
| GT28 | 1 | 1 | 1 |
| GT35 | 1 | 1 | 2 |
| GT39 | 1 | 1 | 1 |
| GT51 | 2 | 2 | 1 |
| GT94 | 1 | 0 | 1 |
| NC | 1 | 2 | 1 |
