# Supplementary material for: The Genome Sequences of Cellulomonas fimi and “Cellvibrio gilvus” Reveal the Cellulolytic Strategies of Two Facultative Anaerobes, Transfer of “Cellvibrio gilvus” to the Genus Cellulomonas, and Proposal of Cellulomonas gilvus sp. nov
Source: PLoS One. 2013 Jan 14;8(1):e53954. doi: 10.1371/journal.pone.0053954 (PMC3544764; doi:10.1371/journal.pone.0053954)
Supplement: Table S3 — Secreted CAZymes in the sequenced cellulomonads. (DOC) [file pone.0053954.s006.doc]

**Table S3. Secreted CAZymes in the sequenced cellulomonads^α^**.

| **Statistic** | ***Cellvibrio gilvus*** | ***Cellulomonas flavigena*** | ***Cellulomonas fimi*** |
| --- | --- | --- | --- |
| Total CAZymes | 126 (50.0%) | 155 (48.4%) | 173 (45.1%) |
| Sec-dependent secretion | 43 (34.1%) | 56 (36.1%) | 52 (30.1%) |
| Tat-dependent secretion | 20 (15.9%) | 19 (12.3%) | 26 (15.0%) |
| Transmembrane segment | 5 | 8 | 13 |

^α^ Predicted Carbohydrate Active enzymes (CAZymes) were obtained from the CAZyme database (www.cazy.org) and secreted enzymes were predicted using the PRED-TAT software . Parentheses indicate percent of total CAZymes that are secreted.
