# Supplementary material for: The Genome Sequences of Cellulomonas fimi and “Cellvibrio gilvus” Reveal the Cellulolytic Strategies of Two Facultative Anaerobes, Transfer of “Cellvibrio gilvus” to the Genus Cellulomonas, and Proposal of Cellulomonas gilvus sp. nov
Source: PLoS One. 2013 Jan 14;8(1):e53954. doi: 10.1371/journal.pone.0053954 (PMC3544764; doi:10.1371/journal.pone.0053954)
Supplement: Table S4 — Cellulases, Xylanases, and hemicellulases of the sequenced cellulomonads. (DOC) [file pone.0053954.s007.doc]

**Table S4. Cellulases (Yellow), Xylanases (Green), and hemicellulases (Purple) of the sequenced cellulomonads.**

|  | ***C. gilvus*** | ***C. fimi*** | ***C. flavigena*** |
| --- | --- | --- | --- |
| **Cellulases** |  |  |  |
| **GH5 Total** | **2** | **3** | **3** |
| GH5 |  |  | cfla_0736(s) |
| GH5 CBM2 | celgi_1696(s)  celgi_2637(s) | celf_1924(s) | cfla_1897(s)  cfla_2811(s) |
| GH5 CBM13 |  | celf_2403(s) |  |
| GH5 CBM46 |  | celf_0376(s) |  |
| **GH6 Total** | **4** | **4** | **3** |
| GH6 | celgi_2723(s)  celgi_2269(s) | celf_0233(t)  celf_1230(s) |  |
| GH6 CBM2 | celgi_1697(t)  celgi_2718(t) | celf_1925(s)  celf_3184(t) | cfla_1896(s)  cfla_2912(t)  cfla_2913(s) |
| **GH9 Total** | **2** | **4** | **5** |
| GH9 |  | celf_1481* |  |
| GH9 CBM3 CBM2 | celgi_0019(t) | celf_0019(s) | cfla_0016(t)  cfla_0139(s) |
| GH9 CBM2 |  | celf_0045(s) | cfla_3563(t) |
| GH9 CBM4 CBM4 | celgi_2074(s) | celf_1705(t) | cfla_1515(s)  cfla_3031(s) |
| **GH48 Total** | **1** | **1** | **1** |
| GH48 CBM2 | celgi_2669(s) | celf_3400(t) | cfla_3105(t) |
| **GH94 Total** | **1** | **1** | **1** |
| GH94 | celgi_3055* | celf_0317* | cfla_0148* |
| **Xylanases** |  |  |  |
| **GH10 Total** | **6** | **5** | **16** |
| **GH10** | celgi_1889* | celf_1729* | cfla_0809  cfla_2048  cfla_2327  cfla_3547 |
| GH10 CBM2 | celgi_1040  celgi_3149 | celf_0088  celf_1271 | cfla_0809  cfla_2048  cfla_2327  cfla_3547 |
| GH10 CBM13 | celgi_1915 | celf_3156 | cfla_0376 |
| GH10 CBM2 CBM22 | celgi_0632 |  | cfla_0905 |
| GH10 CBM2 GH62 |  |  | cfla_3024 |
| GH10 CBM2 GH10 |  |  | cfla_2487  cfla_2558 |
| GH10 CBM22 CBM22 CBM9 CE4 | celgi_2403 | celf_0574 |  |
| GH11 CBM2 CE4 GH10 CBM2 |  |  | cfla_0245 |
| CBM22 CBM22 CBM22 CE4  CBM22 GH10 GH9 |  |  | cfla_0638 |
| CBM22 CBM22 GH10 GH9 |  |  | cfla_0639 |
| **GH11 Total** | **1** | **1** | **3** |
| GH11 CBM2 CE4 CBM2 | celgi_3003 | celf_0374 | cfla_0244 |
| GH11 CBM2 CE4 GH10 CBM2 |  |  | cfla_0245 |
| GH11 CBM2 |  |  | cfla_0246 |
| **GH30 Total** | **1** | **1** | **0** |
| GH30 | celgi_0814 |  |  |
| GH30 CBM13 |  | celf_3633 |  |
| ***β*-xylosidases, *α*-arabinofuranosidases, and *α*-glucuronidases** | | | |
| **GH43 Total** | **7** | **7** | **5** |
| GH43 | celgi_2558*  celgi_3055*  celgi_0158  celgi_2552  celgi_2571  celgi_2586  celgi_2589 | celf_1745*  celf_0899*  celf_3643*  celf_0788  celf_0904  celf_1482 | cfla_0551*  cfla_3048* |
| GH43 CBM13 |  | celf_3157 | cfla_1280 |
| GH43 CBM2 |  |  | cfla_0179 |
| GH43 CBM6 CBM13 |  |  | cfla_0455 |
| **GH51 Total** | **2** | **4** | **1** |
| GH51 | celgi_2553*  celgi_2572 | celf_0903*  celf_1746*  celf_3249*  celf_3321* | cfla_3528* |
| **GH62 Total** | **1** | **1** | **2** |
| GH62 CBM13 | celgi_2680 | celf_3155 | cfla_2848 |
| GH62 GH10 CBM2 |  |  | cfla_3024 |
| **GH67 Total** | **0** | **1** | **1** |
| GH67 |  | celf_3268* | cfla_2984* |
| **GH120 Total** | **0** | **1** | **1** |
| GH120 |  | celf_1752* | cfla_0468* |
| **GH121 Total** | **1** | **1** | **0** |
| GH121 | celgi_2587* | celf_0787 |  |

*no predicted signal peptide detected

(s) = sec signal peptide detected

(t)= tat signal peptide detected
